# Cost-effectiveness of early initiation of first-line combination antiretroviral therapy in Uganda

**DOI:** 10.1186/1471-2458-12-736

**Published:** 2012-09-04

**Authors:** Joseph Sempa, Mark Ssennono, Andreas Kuznik, Mohammed Lamorde, Stefanie Sowinski, Aggrey Semeere, Sabine Hermans, Barbara Castelnuovo, Yukari C Manabe

**Affiliations:** 1 Infectious Diseases Institute, College of Health Sciences, Makerere University, P.O. Box 22418, Kampala, Uganda; 2 Pfizer Inc., New York, NY, USA; 3 Department of Pharmacology and Therapeutics, Trinity College, Dublin, Ireland; 4 Gladstone Institute of Virology and Immunology, 1650 Owens Street, San Francisco, CA, 94158, USA; 5 Department of Internal Medicine and Infectious Diseases, University Medical Center Utrecht, Utrecht, the Netherlands; 6 Department of Medicine, Division of Infectious Diseases, Johns Hopkins School of Medicine, Baltimore, MD, USA

**Keywords:** HAART, Economics, Mortality, Adverse effects

## Abstract

**Background:**

Ugandan national guidelines recommend initiation of combination antiretroviral therapy (cART) at CD4^+^ T cell (CD4) count below 350 cell/μl, but the implementation of this is limited due to availability of medication. However, cART initiation at higher CD4 count increases survival, albeit at higher lifetime treatment cost. This analysis evaluates the cost-effectiveness of initiating cART at a CD4 count between 250–350 cell/μl (early) versus <250 cell/μl (delayed).

**Methods:**

Life expectancy of cART-treated patients, conditional on baseline CD4 count, was modeled based on published literature. First-line cART costs $192 annually, with an additional $113 for patient monitoring. Delaying initiation of cART until the CD4 count falls below 250 cells/μl would incur the cost of the bi-annual CD4 count tests and routine maintenance care at $85 annually. We compared lifetime treatment costs and disability adjusted life-expectancy between early vs. delayed cART for ten baseline CD4 count ranges from 250-350 cell/μl. All costs and benefits were discounted at 3% annually.

**Results:**

Treatment delay varied from 6–18 months. Early cART initiation increased life expectancy from 1.5-3.5 years and averted 1.33–3.10 disability adjusted life years (DALY’s) per patient. Lifetime treatment costs were $4,300–$5,248 for early initiation and $3,940–$4,435 for delayed initiation. The cost/DALY averted of the early versus delayed start ranged from $260–$270.

**Conclusions:**

In HIV-positive patients presenting with CD4 count between 250-350 cells/μl, immediate initiation of cART is a highly cost-effective strategy using the recommended one-time per capita GDP threshold of $490 reported for Uganda. This would constitute an efficient use of scarce health care funds.

## Background

Currently, combination antiretroviral therapy (cART) is the only effective treatment for HIV. This therapy reduces HIV-related morbidity and mortality in the individual patient and can also prevent transmission of HIV to the uninfected population [1-4]. Although cART is clearly beneficial, access to this treatment is limited by a lack of resources, particularly in low-income countries. In western countries, treatment guidelines recommend treatment initiation when severe immune suppression has not yet occurred, with some current guidelines recommending a threshold below 500 cells/μl for initiating cART [5,6].

Despite the evidence of the benefits of early initiation of ART [7,8]in sub-Saharan Africa, where the HIV pandemic is most severe, cART is usually commenced at later stages of the disease and often after the onset of Acquired Immune Deficiency Syndrome (AIDS) [9] when the risk of death is much higher [10].

In 2010, the World Health Organization (WHO) recommended earlier initiation of cART for HIV-infected patients in developing countries [11]. The immunologic threshold for cART initiation was increased from a CD4^+^ T cell (CD4) count of 200 cells/μL to 350 cells/μL, effectively increasing the number of patients eligible for cART. Importantly, at the end of 2010, only 47% of eligible patients in developing countries were actually accessing cART based on the prior WHO cut-off of 350 cells/μL [12]. In Uganda, where approximately 1.2 million people are infected with HIV, WHO recommendation were adopted and the national guidelines recommend to increase the threshold from 250 to 350 cells/μL [13]. However, in 2009, only 53.5% of Ugandan adults with CD4 count below 250 cells/μL were receiving cART [14] and with the current WHO and National recommended ART eligibility criteria of CD4 count < 350 cell/μL [11,13],the coverage of ART drops to 47% [13]. Additionally, adoption of the 2010 revised WHO guidelines is expected to increase the number of patients on cART and the work load of HIV programs, and consequently increase costs associated with cART provision [15]. In general, the adoption of the WHO recommendations seems to be delayed in countries with relatively low health expenditure per capita and as well as low gross domestic product per capita, and costs associated with availability of cART is often cited as a reason for delayed initiation of cART [16]. In resource-constrained settings, like Uganda, evidence of the cost-effectiveness of these new guidelines is needed to inform policy and to support decision-making in the allocation of scarce health care resources. We aimed to compare the cost-effectiveness of initiating cART in patients using the revised CD4 count threshold of 350 cells/μL as in the WHO 2010 guidelines versus (vs) cART initiation using a threshold of 250 cells/μL.

## Methods

### Model overview

A decision analytic model was used to calculate the disability adjusted life expectancy for patients with CD4 count between 250 and 350 cells/μL under two treatment scenarios: A, initiate cART immediately and B, hold cART initiation until the CD4 count falls below 250 cells/μL. In our analysis, we used the standard cost per disability adjusted life years (DALY’s) metric. The DALY is defined as the sum of years of life lost (YLL), and years lived with disability (YLD) [18]. Net costs were defined as the sum of discounted lifetime treatment cost associated with cART, including drug and patient monitoring costs, and the hospitalization costs with and without severe opportunistic infections within the first 24 months after initiation of therapy. Based on published sources, we calculated the life expectancy of patients initiating cART with baseline CD4 count between 250 cells/μL and 350 cells/μL. Patients who initiated treatment immediately (Scenario A) were assigned a life expectancy [19]; patients who initiated cART after their CD4 count fell below 250 cells/μL (Scenario B) were assigned another lower life expectancy which we defined as the sum of the waiting time plus the life expectancy associated with the lower baseline CD4 count at initiation of therapy [19] (see more detailed explanations of the model inputs and outputs below). Select inputs were varied in one-way sensitivity analyses (see Table 1) and all calculations were performed in Excel 2007 (Microsoft Corporation, Redmond, WA, USA). 

**Table 1:** Model Inputs for the patient characteristics and cost

**Input**	**Value**	**Sensitivity Range**		**Source Data**
Life Expectancy, in years				
CD4 = 50-200	9.60	8.16	11.04	[19]
CD4 = 200-350	19.30	16.41	22.20	[19]
CD4 tests per year	2			[20]
Median CD4 cell decline, per 6 months	45.75	30.65	62.35	[21]
DALY Adjustment^a^				
HIV	0.123			[22]
AIDS	0.5			[22]
Years of AIDS before death	1			Assumption
Annual Number of Inpatient Days with OI^b,c^				
CD4 > 350 (on cART)	0.37	0.19	0.56	[23]
CD4 > 350 (off cART)	5.7	2.85	8.55	[23]
CD4 = 201-350 (on cART)	0.52	0.26	0.78	[23]
CD4 = 201-350 (off cART)	10.8	5.4	16.2	[23]
Annual Number of Inpatient Days without OI^b,c^				
CD4 > 350 (on cART)	0.14	0.07	0.21	[23]
CD4 > 350 (off cART)	1.9	0.95	2.85	[23]
CD4 = 201-350 (on cART)	0.39	0.2	0.59	[23]
CD4 = 201-350 (off cART)	3	1.5	4.5	[23]
Costs				
cART drug cost, per year^d^	$192.44	$100	$300	[24]
cART maintenance cost, year	$113.40	$50	$200	[25]
Total Treatment cost, sum	$305.84	$150	$500	[24,25]
Annual costs incurred while waiting				
Clinic Personel	$14.32			[25]
Lab	$35.04			[25]
Other Medication	$33.72			[25]
Radiology	$1.68			[25]
Sum	$84.76	$0	$150	[25]
Cost per Inpatient Day	$31.48			[26]
Discount Rate	3%	0%	6%	[18]

^a^DALY - Disability Adjusted Life Years.

^b^ OI – Opportunistic infections.

^c^ The sensitivity range is not reported in [23]. In our model, we assume a 50% decrease and 50% increase in OI’s relative to the base case.

^d^cART- combination antiretroviral therapy, containing: AZT – zidovudine ; 3TC – lamivudine; EFV- efavirenz.

### Model inputs

**Figure 1:**
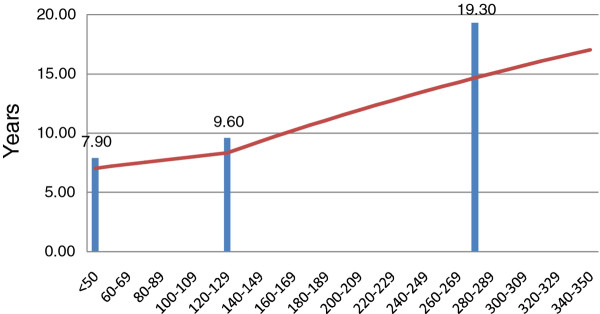
Modeled Life Expectancy by baseline CD4 cell count at initiation of therapy, in years*. *The bars represent published life expectancy data from [18]. **Note: Our continuous life expectancy curve does not intersect the three bars because we apply a 3% discount rate.

Model inputs used in this analysis are displayed in Table 1. Estimates derived from a systematic analysis using Markov modeling of cART survival data for a hypothetical Tanzanian population from 20 observational studies and cohort analyses serve as the basis for our modeling of conditional life expectancies in Uganda [19]. We used this approach as similar life expectancy estimates are not available for Uganda and the two countries are fairly comparable with respect to a number of health related parameters such as life expectancy at birth and HIV prevalence [27]. In the Tanzanian analysis, life expectancy of patients was positively related to baseline CD4 count at the time of therapy initiation, even after taking into consideration the lead-time bias. Patients who initiated cART at different CD4^+^ T cell count levels, 200 cells/μL −350 cells/μL, 50 cells/μL −199 cells/μL and <50 cells/μL, had an life expectancy of 19.3, 9.6 and 7.9 years, respectively [19]. In our model, we subdivided the baseline CD4^+^ T cellrange of interest (250–350) into 10 more narrow uniformly distributed intervals, ranging from 250–259 cells/μL to 340-350 cells/μL. Assuming that the relationship between baseline CD4^+^ T cell count and life expectancy is linear and assuming that the three life expectancy point estimates apply to the midpoints of these ranges (e.g. 275 cells/μL, 125 cells/μL, and 25 cells/μL), we calculated life expectancies for patients in 10 cells/μL increments between 50-59and 340-350by linear extrapolation (see Figure 1). For patients in scenario B, who are not immediately initiated on cART, we assumed a median annual CD4 count reduction of 91.5 cells/μL [21], with an inter-quartile range (IQR) from 61.3 cells/μL to 124.7 cells/μL. Since CD4 count is generally measured twice per year in resource-limited settings like Uganda [20], this translates into a median biannual CD4 count reduction of 45.8 cells/μL (IQR: 30.7-62.4 cells/μL) in our model. Furthermore, we assumed that patients not meeting the 250 cells/μL threshold would have to wait six months until the next CD4 count measurement. Upon initiation of cART, immune reconstitution in the first year was assumed to occur at a rate of 114 cells/μL per year [21]. 

The burden of opportunistic infections (OIs) and non-AIDS diseases in our study population was based on an analysis conducted among HIV-positive patients in southern Africa [23]. In this study, the average annual number of inpatient days with and without severe opportunistic disease were reported for HIV-positive patients conditional on their CD4 count (200–350 cells/μL; and greater than 350 cells/μL) and conditional on treatment with cART (see Table 1). The annual number of expected inpatient days in our scenario A vs scenario B was calculated for a period of two years in four 6-month intervals and multiplied by the average cost per bed day in a tertiary level hospital, including food and drugs, which for Uganda is reported at $31.5 per day [26]. All cost used in our analysis were computed in 2011 United States dollars ($).

The cost of first-line cART in Uganda, consisting of zidovudine, lamivudine and either efavirenz or nevirapine was estimated at $192 per year [28]. The annual costs of healthcare-related services associated with patient monitoring while on cART were obtained from published sources [28], and included clinic personnel (based on monthly office visits): $43.0, laboratory costs: $35.0, other medications: $33.7, and radiology: $1.7. Based on these costs, we calculated the total annual cost to the health care system for a patient who is initiated on cART at $306. In comparison, HIV-positive patients with CD4 count exceeding the current limit of 250 cells/μL are also routinely followed up and incur annual costs including clinic personnel (based on quarterly office visits): $14.3, laboratory costs: $35.0, other medications: $33.7, and radiology: $1.7. Thus, the average annual costs of waiting to qualify for cART are estimated at $84.8. Lifetime HIV treatment costs were therefore defined as the sum of 1) lifetime first line cART, 2) lifetime patient monitoring, and 3) the cost of OI related hospitalizations in the first 2 years after therapy initiation. A disability weight of 0.1 was used for each year lived with HIV and a disability weight of 0.5 was used for the last year of life with AIDS [22]. All costs and benefits were discounted at 3% annually [18].

### Model structure

**Figure 2:**
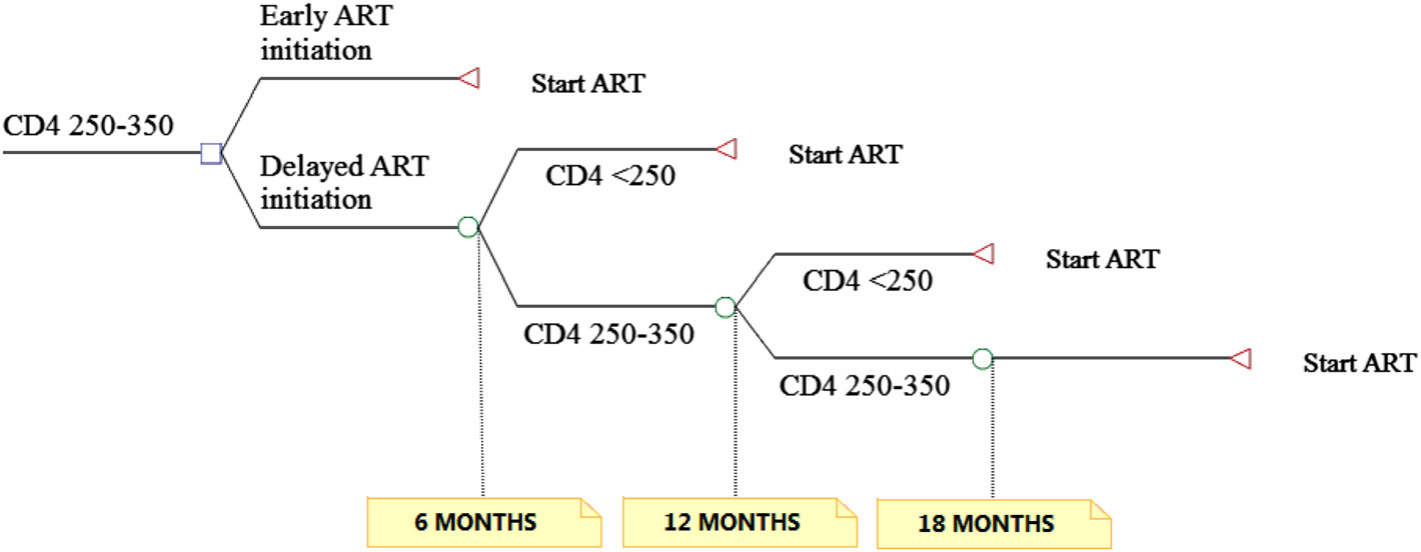
Structure of the model.

In our model, we calculated the life expectancy conditional on baseline CD4 count for the ten CD4 count increments from 250–259 cells/μL to 340–350 cells/μL. For patients in scenario A, these represent their discounted life expectancies. Patients in scenario B were assigned another, lower, life expectancy which we defined as the sum of the waiting time plus the life expectancy associated with the lower baseline CD4 count at initiation of therapy. We calculated their baseline CD4 count at cART initiation by subtracting 45.8 cells/μL per 6 month until their CD4 count fell below the threshold of 250 cells/μL. For example, a patient in scenario B with a CD4 count of 265 cells/μL would have to wait 6 months and start therapy at about 219 cell/μL. His life expectancy is therefore 6 months plus his life expectancy associated with initiation of cART at 219 cells/μL. However, as shown above, 25% of patients in our model experience either a slower or faster CD4 count decline. Therefore, we varied the time to initiation of cART used in our model by the lowest (25th percentile), mean (50^th^percentile) and highest (75th percentile) CD4 count decline rates. We then calculated YLL as the difference in the discounted life expectancies between the two groups, whereas YLD were calculated as the difference in disability weighted life expectancy between the two groups. Finally, the difference in lifetime treatment costs between the two scenarios was computed as the difference in the respective sums of discounted cART drug and monitoring costs, OI and non-AIDS related hospitalization costs (see Figure 2).

## Results

### Life expectancy

Model results are displayed in Table 2. In HIV-positive patients with a baseline CD4 count ranging from 250–350 cells/μL, mean life expectancy with immediate initiation of cART (Scenario A) was approximately 20.9 years (discounted: 14.7 years), and varied from as low as 18.0 years (discounted: 13.9 years) to as high as 23.9 years (discounted: 17.0 years).

**Table 2:** Projected estimates associated with each scenario

	**Baseline CD4 count**	**Start (Scenario A)**	**Wait (Scenario B)**
Life Expectancy, in years	Lowest (250–259)	18.01	15.52
	Mean (250–350)	20.93	16.96
	Highest (340–350)	23.85	17.38
Discounted Life Expectancy, in years	Lowest (250–259)	13.91	12.41
	Mean (250–350)	15.51	13.29
	Highest (340–350)	17.04	13.54
YLL	Lowest (250–259)		1.50
	Mean (250–350)		2.23
	Highest (340–350)		3.49
YLD	Lowest (250–259)		−0.17
	Mean (250–350)		−0.25
	Highest (340–350)		−0.39
DALY	Lowest (250–259)		1.33
	Mean (250–350)		1.98
	Highest (340–350)		3.10
Lifetime Cost, cART + Monitoring	Lowest (250–259)	$4,255	$3,686
	Mean (250–350)	$4,744	$3,885
	Highest (340–350)	$5,210	$3,870
24 Months Hospitalization Cost	Lowest (250–259)	$45	$254
	Mean (250–350)	$42	$389
	Highest (340–350)	$38	$565
Net Cost	Lowest (250–259)	$4,300	$3,940
	Mean (250–350)	$4,786	$4,274
	Highest (340–350)	$5,248	$4,435
Cost per DALY	Lowest (250–259)	$270	
	Mean (250–350)	$260	
	Highest (340–350)	$262	

cART- combination antiretroviral therapy; YLD – Years Lost due to Disability; DALY - Disability Adjusted Life Years.

Based on current guidelines, HIV-positive patients would have to wait until their CD4 count falls below the threshold of 250 cells/μL (Scenario B). In our model, the waiting time until initiation of cART for patients with CD4 count above 250 cells/μL but below 350 cells/μL ranged from a minimum of 6 months to a maximum of 18 months. By the time cART was initiated, the median CD4 count in this group had fallen to 226 cells/μL (IQR:216–234 cells/μL). The ensuing mean life expectancy of 17.0 years (discounted: 13.3 years) varied from a low of 15.5 years (discounted: 12.41 years) to a high of 17.4 years (discounted: 13.5 years). Our model found that the waiting strategy (scenario B) was associated with an average of 2.2 YLL lost, which translated into 1.98 DALY’s.

In our model, all patients in scenario B initiated therapy within 6–18 months from the date of their first CD4 count record ranging between 250 cells/μL −350 cells/μL. However, by the time of cART initiation, the net life expectancy associated with the now lower CD4 count decreased by a discounted 2.0 – 4.7 years. While the cost of antiretroviral drugs was deferred temporarily, some monitoring costs were still incurred. In addition, 35%-40% of these short-term savings were offset by increases in the costs of treating opportunistic infections.

### Treatment costs and cost-effectiveness

Mean lifetime HIV drug and monitoring costs for patients in Scenario A were $4,744 and ranged from a low of $4,255 for patients in the lowest CD4 count range of 250–259 cells/μL to $5,210 for patients in the highest CD4 count range of 340–350 cells/μL. The mean cost of hospitalizations in the first 24 months with and without severe opportunistic infections in this group accounted for an additional $42 (range: $38 to $45), yielding net lifetime costs of $4,786 (Range: $4,300 to $5,248). In comparison, Scenario B patients incurred lifetime HIV drug and monitoring costs of $3,885 (range: $3,686 to $3,870) and hospitalization costs of $389 (range: $254 to $565), yielding net costs of $4,274 (range: $3,940 to $4,435). Thus, in patients with a CD4 count of 250–350 cells/μL, immediate initiation of cART versus waiting until they meet the current CD4 count threshold of 250 cells/μL was expected to increase lifetime treatment costs by an average of $512 (Range: $360 to $814) and was associated with a cost/DALY of $260 (range: $262-$270).

### Sensitivity analyses

**Figure 3:**
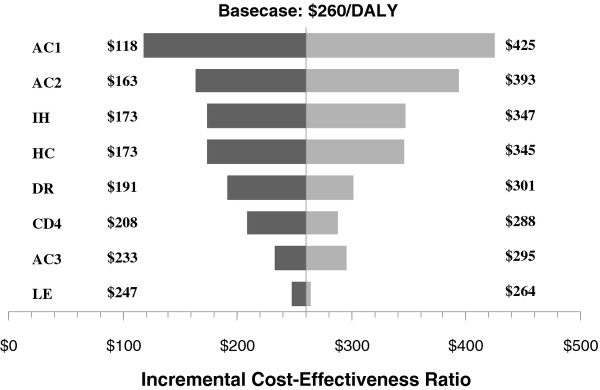
**One-way Sensitivity Analyses of Key Parameters (Basecase Values).** AC1: Annual cost of cART ($192.44): $100-$300; AC2: Annual cost of cART maintenance ($113.40): $50-$200; IH: Number of inpatient hospitalizations (as per Bendavid et al., Ref: [22]): -50%/+50%; HC: Daily Hospitalization Cost ($31.48): $15.74-$47.22; DR: Discount Rate (3%): 0%-6%; CD4: Average CD4 decline in 6 months (45.75): 30.65-62.35; AC3: Annual costs of patient monitoring while not yet on cART ($84.76): $0-$150; LE: Remaining life expectancy (as per Johansson, Ref: [18]): -15%/+15%.

We used the mean cost/DALY of $260 as the base-case for the sensitivity analyses. Varying select model inputs had no discernible impact on our cost/DALY estimate. The results obtained in the various one-way sensitivity analyses are displayed in the tornado diagram in Figure 3. The incremental cost-effectiveness ratio(ICER) was most sensitive to the annual cost of ART and annual cost of ART maintenance. The number of hospitalizations, daily hospitalization costs, discount rate, CD4 count average decline, annual cost of patient monitoring, and remaining life expectancy did not have a significant impact on the ICER.

## Discussion

To our knowledge, this is the first study to evaluate the cost-effectiveness of early cART initiation taking in account that in resource-limited settings pre-cART CD4 count measurements frequency (every 6 months). We incorporated the usual waiting time in Uganda into a decision analytic framework to model the clinical and economic impact of a hypothetical choice: whether or not to initiate cART immediately, in this patient population with CD4 counts between 250 and 350 cells/μL.

Our model shows that initiation of cART among patients with higher CD4 count (250 – 350 cells/μL) is associated with an average cost per DALY averted of $260. According to WHO cost-effectiveness analysis guidelines for resource-constrained settings, an intervention is highly cost-effective if it is associated with a cost/DALY less than one time per capita GDP [18]. Annual per capita GDP in Uganda is $490 per year [28]. Thus, we conclude that early initiation of first-line cART among patients with CD4 count from 250 – 350 cells/μL is highly cost-effective.

Comparable studies in this area have simulated HIV disease progression using Markov models, were generally conducted in higher cost settings within sub-Saharan Africa, and were often conducted at times when antiretroviral drugs were still priced higher than they are today. Nevertheless, these studies also found that it is cost-effective to initiate therapy at CD4 count below 350 cells/μL compared to 200 or 250 cells/μL, with an increase in life expectancy ranging from 0.8-2.3 years (discounted), an incremental lifetime cost of $ 924-$8936 and a cost/QALY of $ 766-$3885 [29-32]. In comparison to these previous studies, our results present greater benefits and lower costs associated with earlier commencement of cART, in part, due to ongoing reductions in the cost of cART in developing countries [33,34].

There are additional benefits associated with early initiation of cART like a decrease in the HIV transmission risk resulting from a decreased viral load which we did not take into account as recent evidence showed that among discordant couples, cART resulted in a 96% decrease in the risk of horizontal HIV transmission [3]. Thus, by only including health benefits as they apply to the treated patient and ignoring the public health impact of a decrease in the risk of HIV-transmission, our model underestimates the actual cost-effectiveness of earlier commencement of cART. In addition, there is mounting evidence that earlier initiation of cART also averts non-HIV associated morbidity (e.g. cardiovascular and renal events) that we did not factor into our model because data from resource-limited settings has not yet been generated [35,36].

Our findings provide supportive evidence for advocacy and changes in health policy to increase cART funding in order to make this treatment available to a greater number of patients. Currently, in Uganda as well as in other developing countries, cART provision is largely dependent on foreign donors. International funding for HIV programs have stagnated or even decreased in the recent years [37]. However, recent work suggests that the HIV epidemic can be reversed if an investment framework is instituted that involves basic programs including cART provision [38]. While we emphasize that a cost-effective intervention may not necessarily be affordable in an environment where potentially multiple cost-effective interventions compete for scarce resources, this analysis does suggest that an investment in earlier cART initiation would likely constitute a highly cost-effective use of scarce resources. Given the apparent health and economic benefits of earlier initiation of cART, efforts should be intensified to provide increased and sustainable levels of funding to support cART provision.

### Limitations

Our analysis was not without limitations. First, life expectancy estimates on which this analysis is based were not derived from a randomized clinical trial but were obtained from a published analysis that extrapolates annual survival data from 20 observational studies and cohort analyses using Markov modeling and reports life expectancies for three baseline CD4 count categories [19]. Furthermore, in our model we assume a linear relationship between life expectancy and baseline CD4 count over the range of 250–349 cells/μl at the initiation of cART. This might lead to overestimation of the health economic benefit of early cART initiation in patients with CD4 count on the high end of this range if the absolute increase in life-expectancy were to diminish with higher baseline CD4 count. Also, we did not include failure of first-line cART in our model, which would necessitate progression to second-line cART. Second-line antiretroviral drugs are substantially more expensive and may therefore potentially have a significant have an impact on our results. However, there is no evidence to suggest that the rate of first-line treatment failure would be expected to differ between our two treatment arms. In addition, the rate of cART treatment failures requiring a switch to second line therapy is reported to be relatively low in Uganda (<1% annually) [39]. Lastly, we did not include the impact of adherence in our model. Patients starting cART earlier may be asymptomatic and have less incentive to take their medication than patients in whom cART initiation is delayed [40]. Conversely, delaying cART may put patients at risk of severe opportunistic infections which may impair adherence by increasing pill burden or by directly affecting cognitive function. The ultimate impact of adherence on our cost-effectiveness ratio is therefore unclear and further research is warranted.

## Conclusion

In summary, immediate initiation of cART is highly cost-effective in HIV-positive patients presenting with CD4 count between 250–350 cells/μl in Uganda. Expanding the number of treatment slots to include patients with higher CD4 count would be an efficient use of scarce health care funds.

## Competing interests

Andreas Kuznik is a full time employee of Pfizer Inc., with ownership of stock in Pfizer Inc., and his time at the Infectious Diseases Institute in Uganda was supported by the Pfizer Global Health Fellows program.

## Authors’ contribution

JS was responsible for data collection, data interpretation, and literature search; MS was involved in Literature search and data collection; AK was responsible for the study design, data interpretation and developed the cost-effectiveness model. ML also did data collection, model interpretation, and literature search; SS, AS, and SH also participated in literature searches and data collection; BC and YC were also involved in literature searches, data interpretation, and data collection. All authors contributed towards drafting the manuscript and/or revising it for intellectual content and have given final approval of the version to be published.

## Pre-publication history

The pre-publication history for this paper can be accessed here:

http://www.biomedcentral.com/1471-2458/12/736/prepub
